# Spray-Drying Performance and Thermal Stability of L-ascorbic Acid Microencapsulated with Sodium Alginate and Gum Arabic

**DOI:** 10.3390/molecules24162872

**Published:** 2019-08-07

**Authors:** Pabla A. Barra, Katherine Márquez, Oscar Gil-Castell, Javiera Mujica, Amparo Ribes-Greus, Mirko Faccini

**Affiliations:** 1 R&D Department, Leitat Chile, Calle Román Díaz 532, Providencia, Santiago 7500724, Chile; 2 Instituto de Tecnología de Materiales (ITM), Universitat Politècnica de València (UPV), Camino de Vera s/n, 46022 Valencia, Spain; 3 Departament d’Enginyeria Química, Escola Tècnica Superior d’Enginyeria, Universitat de València, Av. de la Universitat, s/n, 46100 Burjassot, Spain; 4 Centro de Excelencia en Nanotecnología (CEN) Chile, Calle Román Diaz 532, Providencia, Santiago 7500724, Chile; 5 Materials Chemistry Division, Leitat Technological Center, C/Pallars 179–185, 08005 Barcelona, Spain

**Keywords:** L-ascorbic acid, sodium alginate, gum arabic, spray-drying, encapsulation

## Abstract

The potential of sodium alginate (ALG) and gum arabic (GA) as wall polymers for L-ascorbic acid (AA) encapsulation as a tool for their preservation against the thermo-oxidative degradation was investigated. The influence of such polymers used as wall material on the AA-content, size, encapsulation efficiency, encapsulation yield and thermo-oxidative stability were evaluated. The AA-microparticles were obtained using the spray-drying technique. An experimental Taguchi design was employed to assess the influence of the variables in the encapsulation process. The microparticles morphology and size distribution were characterized by scanning electron microscopy and laser diffraction. The thermal stability of AA microparticles was studied by differential scanning calorimetry and thermogravimetry analysis. This work points out the viability to encapsulate AA using GA and ALG through a spray-drying process. In general, a product yield ranging from 35.1% to 83.2% and an encapsulation efficiency above 90% were reached. Spherical microparticles with a smooth surface were obtained with a mean diameter around 6 μm and 9 μm for the those prepared with GA and ALG, respectively. The thermo-oxidative analysis showed that both polymers allow maintaining AA stable up to 188 °C, which is higher than the traditional processing temperature used in the fish feed industry.

## 1. Introduction

L-ascorbic Acid (AA), also known as Vitamin C, is a representative water-soluble vitamin and an essential nutrient widely used in fish diets as supplement and antioxidant [1]. It has an important role as cofactor in many biological processes including cellular functions related to neuromodulation, hormone, immune system and collagen synthesis, which is essential to the connective tissues, bone matrix and scar tissue in wound healing [2,3]. Unfortunately, most fish are not capable of biosynthesizing this nutrient due to the absence of the enzyme L-gluconolactone oxidase [2,4]. As a result of vitamin C deficiency, two syndromes have been reported in a variety of different fish: (i) a marked reduction in wound-healing capacity and (ii) skeletal malformation syndrome including mandible and articulation deformity, spinal lordosis, scoliosis and spinal fracture among others [2,3]. Therefore, dietary vitamin C supplementation is required in some fish species and is dependent on the metabolic functions and development stages [5,6].

**Figure 1:**
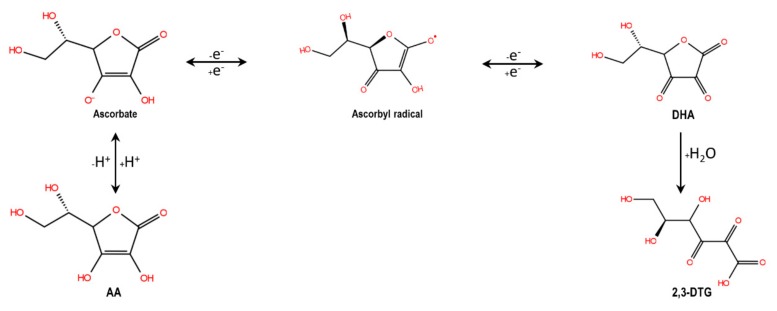
Degradation of ascorbic acid (AA) to dehydroascorbic acid (DHA) and 2,3-diketogulonic acid (2,3-DTG) by UV irradiation by photooxidation and subsequent hydrolysis.

AA is sensitive to light and heat and is degraded to form dehydroascorbic acid and 2,3-diketogulonic acid on UV irradiation by photooxidation and subsequent hydrolysis as shown in Figure 1 [4,7,8,9,10]. Moreover, its rate of oxidation increases in the presence of metals such as copper or iron [11]. This poor stability is a significant problem in the fish feed industry, where the AA is exposed to high temperatures and pressure during the pellet manufacturing process by extrusion [12,13,14]. Phosphate and sulphate derivatives of AA, which are more stable forms of AA, are currently available and have antiscorbutic activity in several aquatic animal species, including L-ascorbyl-2-monophosphate (Mg salt), L-ascorbyl-2-polyphosphate or L-ascorbyl-2-sulfate (K salt) [15]. Nevertheless, the high price of these compounds compared with the AA leads to a significant increase in manufacturing costs [16]. For this reason, it is essential to develop and use cost-effective and viable technologies to increase the stability of ascorbic acid.

Microencapsulation is the most common technology used to maintain the stability of sensitive compounds such as enzymes, pigments, minerals, or vitamins among others. It is a process in which active ingredients are enclosed within a layer of coating/wall polymer to protect them from the external environment [17]. Different approaches have been used to stabilize AA such as fluidized bed coating [18,19], liposomes [20,21], spray chilling [22,23], solvent evaporation [24,25], melt extrusion [26,27], ionic gelation [28,29,30], complex coacervation, [31,32] or spray drying [24,33,34,35,36,37]. Among these methods, spray drying is one of the most reasonably economical, straightforward, continuous and simple to scale-up technique [38,39]. Several research groups have microencapsulated AA by spray drying using different wall materials such as gum arabic [33,40], maltodextrin, modified starches [41], Eudragit [36], concentrated protein isolated and chitosan [35,42] or combinations of such materials [7]. These studies deepened into the evaluation of processing conditions and their effect on microencapsulation efficiency, stability, controlled release and bioavailability of AA. Nevertheless, there is little information available about the thermal and thermo-oxidative stability of microencapsulated AA, which is essential for the designing of a suitable pellet manufacturing process [16,43].

The aim of this study was, therefore, to evaluate and compare the microencapsulation efficiency, the spray-drying performance, the morphological features, the particle size and thermo-oxidative stability of AA microparticles obtained by means of the spray drying technique using sodium alginate and gum arabic as wall materials. These polymers were selected based on their low cost, easy scalability, FDA certification and GRAS category. This work contributes to assess the viability of using microparticles in the pellet manufacturing process for the preparation of supplemented food in the fish-feed industry.

## 2. Materials and Methods

### 2.1. Materials

The L-ascorbic acid (AA) (100% purity), sodium alginate salt from brown algae (ALG) and gum arabic (GA) were supplied by Sigma-Aldrich (Budapest, Hungary). The 2-6-dichlorophenol-indophenol sodium salt glacial acetic acid, metaphosphoric acid and sodium hydrogen carbonate were supplied by Merck Millipore, Darmastardt, Germany. All reagents were of analytical grade and used without any further purification.

### 2.2. Preparation of Microparticles by Spray Drying

The Table 1 gathers the different quantities of compounds used to prepare the microparticles by spray drying. Briefly, the polymer (ALG or GA) was dissolved into 250 mL of distilled water under continuous stirring at 25 °C during 3 h at 1000 rpm for the AA:polymer ratio of 1:1 and 1:2 and at 1500 rpm in the case of AA/polymer ratio of 1:4. Then, under constant stirring, the AA solution was added to the polymer. After 1 h, the sample solution was introduced in the Mini Spray Dryer B-290 BÜCHI (Flawil, Switzerland) with a nozzle size of 0.7 mm and a peristaltic pump at a flow rate of 2–7 (mL·min^−1^). The inlet and outlet temperature used were 140 ± 1 °C and 86 ± 1 °C, respectively. The experiments were performed in triplicates. The spray-dried powders were collected, kept in amber glass bottles and stored in a desiccator until further analyses.

### 2.3. Experimental Taguchi Factorial Design

The statistical analysis was performed with the DOE PRO XL version 3.0 (a Microsoft Excel add-in, available from www.sigmazone.com). The qualities of the fitted models were examined by the coefficient of determination (R^2^). The data were assessed by analysis of variance (ANOVA). A detailed ANOVA framework for assessing the significance of the parameters of the process is also provided (Table S1 in the Supplementary Material).

On the other hand, Taguchi design assesses the variables based on the fractional factorial arrays from the design of experiments (DoE), called orthogonal arrays. Taguchi argues that it is not necessary to consider the interaction between two design variables explicitly, so he developed a system of tabulated design, which reduces the number of experiments as compared to a full factorial design. In this way, Taguchi design was used to evaluate the variables that involved the AA encapsulation. Two different independent variables with three levels each were evaluated. For each variable, the levels were represented by a lower level (−1), medium (0) and higher level (+1), respectively. The values of these levels were chosen according to preliminary experimental studies and literature research [7,8,44,45]. The independent variables were the AA:polymer ratio (1:1; 1:2 and 1:4) and the total dispersed solids (5.0; 12.5 and 20.0 g·L^−1^). The established response variables were the microencapsulation efficiency (%), the mean particle size (µm) and the encapsulation yield (%). The design required nine experiments with two repetitions, as shown in Table 1.

### 2.4. Characterization of Microparticles

#### 2.4.1. Microencapsulation Efficiency

The AA content was assessed by means of the Tillman’s method based on the titration principle with modifications (AOAC Method 967.21) [46]. Regarding the microparticles, 20 mg of them were dissolved in 10 mL of distilled water. Then, the glass vials were introduced into an ice-filled bowl with the sonication probe inserted between the upper quarter and half of the solution. The sonication was performed in four cycles of 1 min on/off using a probe sonicator model Vibra Cell VCX 130, net power output 130W; frequency 20kHz; amplitude 80%, probe 6 mm (Sonics & Materials, Inc. Newtown, CT, USA). The experiments were performed in triplicates and the microencapsulation efficiency was calculated as described in the Equation (1).

(1) $$\mathrm{Microencapsulation}\;\mathrm{Efficency}\;\left(\%\right)=\;\frac{\mathrm{Theoretical}\;\mathrm{AA}\;\mathrm{loading}-\mathrm{unloading}\;\mathrm{AA}}{\mathrm{Theoretical}\;\mathrm{AA}\;\mathrm{concentration}}\times 100$$

#### 2.4.2. Moisture Determination

The moisture content of the microparticles was measured in a Mettler Toledo Model HE73 moisture analyser (Greifensee, Switzerland) at 105 °C immediately after spray-drying. Duplicate samples (0.8 g) of the microparticles were analysed and the mean value was calculated.

#### 2.4.3. Particle Size Distribution

The particle size distribution of the microparticles was carried out by laser diffraction using a Malvern Instruments Mastersizer 3000 (Malven Instruments Ltd, Worcestershire, UK). The samples of powders were dispersed using the Hydro SM small volume sample dispersion accessory. The obscuration was in the interval of 0.5% to 5%. The particle size distribution and corresponding average values were based on at least ten runs for each sample.

#### 2.4.4. Morphological Analysis

The morphological analysis was performed by means of a Zeiss Ultra 55 field emission scanning electron microscope (Carl Zeiss, Thornwood, NY, USA). The samples were placed on metal studs and sputter-coated with a platinum layer during 10 s using a Leica EM MED020 sputter coater (Leica Microsystems, Inc. Buffalo Grove, IL, USA). FE-SEM images were taken at 22 °C with a 1 kV voltage. The microparticles diameters were measured from the scanning electronic microscope images (10,000×) at random locations (*n* = 100) with the Image J^®^ software (Rasband, W.S., ImageJ, U.S. National Institutes of Health, Bethesda, Maryland, USA, https://imagej.nih.gov/ij/, 1997–2018).

#### 2.4.5. Water Activity

The water activity (*a_w_*) measures the availability of water in a food product. The concept of a_w_ is of great importance when determining the quality and safety of a given product. The a_w_ may be defined as the ratio of the vapour pressure of water in food to the vapour pressure of pure water at the same temperature. The AA microparticles a_w_ were measured at temperature 25.0 ± 0.3 °C using an AquaLab Pre Water Activity Analyser (DECAGON Devices. Inc, Pullman, WA, USA).

#### 2.4.6. Structural Analysis

The structural analysis of the pure components and microparticles was assessed by means of Fourier transformed infrared spectroscopy (FTIR) using a Spectrum Two (PerkinElmer Inc., Shelton, CT, USA). The samples were previously ground and mixed thoroughly with potassium bromide at 1:2 (sample:KBr) ratio. The KBr discs were prepared by compressing the powders under a force of 5 Torr for 5 min in a hydraulic press. Sixty-four scans were obtained at a resolution of 2 cm^−1^, from 4500–400 cm^−1^.

#### 2.4.7. Thermo-Oxidative Stability

The thermo-oxidative stability analysis was performed by means of a Mettler-Toledo TGA 851 thermogravimetric analyser (Mettler-Toledo Inc., Schwerzenbach, Switzerland). The samples, with a mass of about 4 mg were introduced in perforated alumina crucibles, with a capacity of 70 μL. The samples were analysed in the temperature range from 25 to 800 °C with a heating rate of 10 °C·min^−1^ under an atmosphere of oxygen at a flow rate of 50 mL·min^−1^. The experiments were performed in triplicates to ensure reproducibility.

#### 2.4.8. Thermal Properties

The calorimetric data were obtained by means of a Mettler-Toledo DSC 820^e^ differential scanning calorimeter (Mettler-Toledo Inc., Schwerzenbach, Switzerland), previously calibrated following the procedure of In and Zn standards. The samples with a mass of about 4 mg were analysed between 0 and 80 °C with a heating/cooling/heating rate of 10 °C·min^−1^. All the experiments were run under nitrogen atmosphere at 50 mL·min^−1^, using an aluminium pan. The specimens were characterised at least by triplicate and the averages of temperatures and enthalpies were taken as representative values.

## 3. Results and Discussion

### 3.1. Spray-Dried L-ascorbic Acid Microparticles

The AA encapsulation process using ALG and GA as wall polymers was performed through an L9 Taguchi design, according to the Table 1. Table 2 shows the obtained average values of the encapsulation efficiency, the mean particle size, the encapsulation yield and the moisture content of the AA:ALG and AA:GA-based microparticles.

The spray-dried AA microparticles showed high encapsulation efficiencies, above 90% for the AA:ALG and over 82% for the AA:GA. In all cases, the encapsulation efficiency was measured by quantifying the entire amount of AA both inside and in the surface of microparticles according to the employed calculation methodology such as described Nielsen [47], Park et al. [48] and Desai et al. [35]. The particle size and the particle diameter distribution were also assessed. The produced AA:ALG-based microparticles showed a mean particle size between 5.13 µm and 14.09 µm, whilst the AA:GA-based microparticles between 2.88 µm and 9.73 µm.

In terms of encapsulation yield, there is an apparent correlation between the obtained values and the content of total dispersed solids for all the AA:ALG-based microparticles. As the polymer concentration increased in the system, the yield of encapsulation decreased, as shown in Table 2. This effect may be attributed to the viscosity increase of the former alginate solutions. The results showed that the major encapsulation yields were obtained when the system was prepared using a concentration of total dispersed solids of 5 g·L^−1^. On the contrary, the results obtained for AA:GA-based microparticles showed that there is no correlation between the total dispersed solid and the encapsulation yield (%). All the studied systems showed encapsulation yield over 61%, regardless of the total dispersed solid. This result is consistent with that reported in literature, in which the effect of the total solid percentages on the microencapsulation yield is negligible due to the low viscosities of the GA aqueous solutions in the concentration range considered in this study [38].

On the other hand, the moisture content is an important indicator for quality control, which may determine the powder suitability, storage behaviour and stability. The average moisture content of the spray dried AA:ALG microparticles fluctuated between 2.14% to 5.48% and between 1.39% to 5.43% for the AA:GA microparticles. The data shows that the moisture content increased for higher ratios of polymer with respect to AA. This effect was more considerable when ALG was employed due to its intrinsic hydrocolloid nature with high hydrophilicity. In general, the moisture values were lower than 6% regardless the wall polymer considered, which is a good indicator of the low susceptibility to the microorganism proliferation and may suggest a propitious storage [7,38,49].

### 3.2. Experimental Taguchi Design

The encapsulation efficiency, the particle size and the encapsulation yield were studied from the perspective of an experimental Taguchi factorial design. The influence of the different variables on the morphological and physical-chemical properties of the AA microparticles were assessed as a function of the wall polymer employed. Moreover, it is feasible to obtain the variables’ contribution to the studied responses through coefficients of the experimental design response polynomial and the ANOVA analysis. The coefficients and ANOVA analysis obtained from the multiple response regression model for each studied factor are listed in Table 3.

In the case of the AA:ALG systems, the encapsulation yield variable has a high correlation coefficient and low *p* coefficients for both independent variables studied in the model (Table S1). Regarding encapsulation efficiency and mean particle size variables, the results have shown that the correlation as well as *p* coefficients do not consider significant (*p* > 0.05). These results confirm that the model is reliable just for describing the encapsulation yield, while for describing the other variables the prediction from the model it is not representative.

On the other hand, for the AA:GA encapsulation the Taguchi design only describing the size particle is significant since for this variable the analysis DoE generates a low p coefficient in both independent variables considered in the model and a high correlation factor (>0.8). For the other dependent variables studied, the model it is not representative.

### 3.3. Morphological Analysis

**Figure 2:**
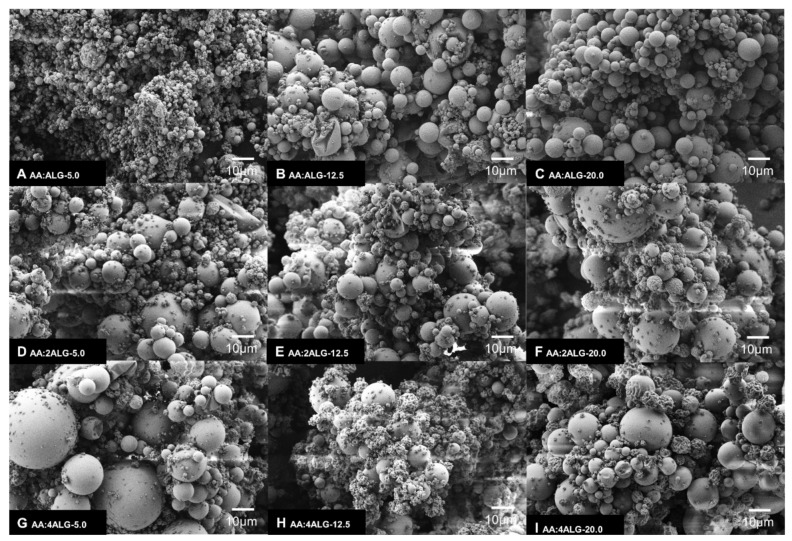
FE-SEM images of AA:ALG-based microparticles from 5 g L^−1^ to 20 g·L^−1^ of total dispersed solids and 1:1, 1:2 to 1:4 AA:ALG-polymer ratio; (**A**) AA:ALG-5.0, (**B**) AA:ALG-12.5, (**C**) AA:ALG-20.0, (**D**) AA:ALG-5.0, (**E**) AA:2ALG-12.5, (**F**) AA:2ALG-20.0, (**G**) AA:4ALG-5.0, (**H**) AA:4ALG-12.5, (**I**) AA:4ALG-20.0.

The obtained microparticles were morphologically analysed by means of field-emission scanning electron microscopy (FE-SEM). Figure 2 shows the representative electronic micrographs of the obtained AA:ALG-based microparticles elaborated for all range of concentrations.

In general, the AA:ALG-based microparticles revealed a spherical geometry and a regular shape without fissures. The figures also demonstrated that when increasing the ALG ratio from 1 to 4, the surface of the microparticles tended to wrinkle, given the incomplete atomization caused by the high viscosity of the solution (Figure 2H,I).

According to the mean size diameter results achieved by Mastersizer (Table 2), the micrographs revealed that most of the microparticles presented a particle size in the range from 5 to 14 µm. Moreover, the FE-SEM images showed that as the ALG ratio increased the AA microparticles exhibited a wider size range, probably due the high viscosity of the solution (Figure 2G–I). Moreover, the variety in size is a typical characteristic of particles produced by means of the spray-drying technique. In summary, the analysis showed that the AA:ALG-5 system presents a homogeneous particle size in comparison to the other systems studied.

**Figure 3:**
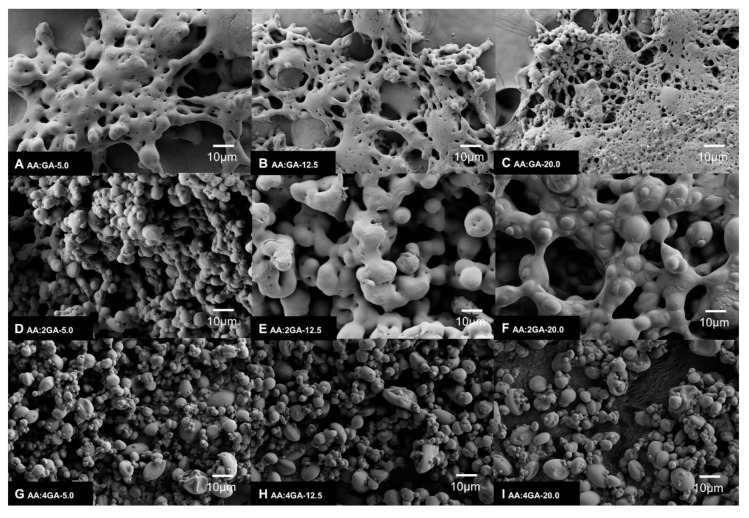
FE-SEM images of AA:GA-based microparticles from 5 g·L^−1^ to 20 g·L^−1^ of total dispersed solids and 1:1, 1:2 to 1:4 AA:GA-polymer ratio; (**A**) AA:GA-5.0, (**B**) AA:GA-12.5, (**C**) AA:GA-20.0, (**D**) AA:2GA-5.0, (**E**) AA:2GA-12.5, (**F**) AA:2GA-20.0, (**G**) AA:4GA-5.0, (**H**) AA:4GA-12.5, (**I**) AA:GA-20.0.

On the other hand, the micrographs of the AA:GA-based microparticles are shown in Figure 3. The FE-SEM images showed that for lower GA content, the microparticles revealed an agglomerated structure without a spherical shape (Figure 3A–C). This deformation was probably due to incomplete atomization because of the action of the viscous forces and surface tension on the bulk liquid. Instead, the microparticles elaborated with polymer ratio above AA:4GA ratio presented a smooth surface with a nearly globular shape and a particle size lower than 10 µm. These observations agree with the results of the mean particle size obtained by Mastersizer analysis where the AA:4GA systems exhibited the most homogeneous particle size regardless the total dispersed solids considered (Figure 3G–I).

### 3.4. Water Activity

Water activity (a_w_) is the amount of unbound water in a sample. This parameter is very relevant to evaluate the safety and quality of the foods since water that is not bound to the ingredients themselves can be used by unwanted microorganisms which could lead to one of the contributing factors for food spoilage. Bacteria usually require at least 0.91 and fungi at least 0.70; hence, keeping low water activity value is usually necessary to inhibit their growth [50].

The results showed that both AA:ALG and AA:GA-based microparticles have a low a_w_ with values of 0.34 in the case of ALG and 0.26 for GA wall polymer (Table S2 in the Supplementary Material). The obtained results indicate that these microparticles do not reach the minimum level of water available required for the growth of microorganisms or bacteria such as *Staphylococcus aureus*, *Salmonella Enteritidis*, *Escherichia coli* O157:H7, and *Campylobacter*.

### 3.5. Structural Characterization

**Figure 4:**
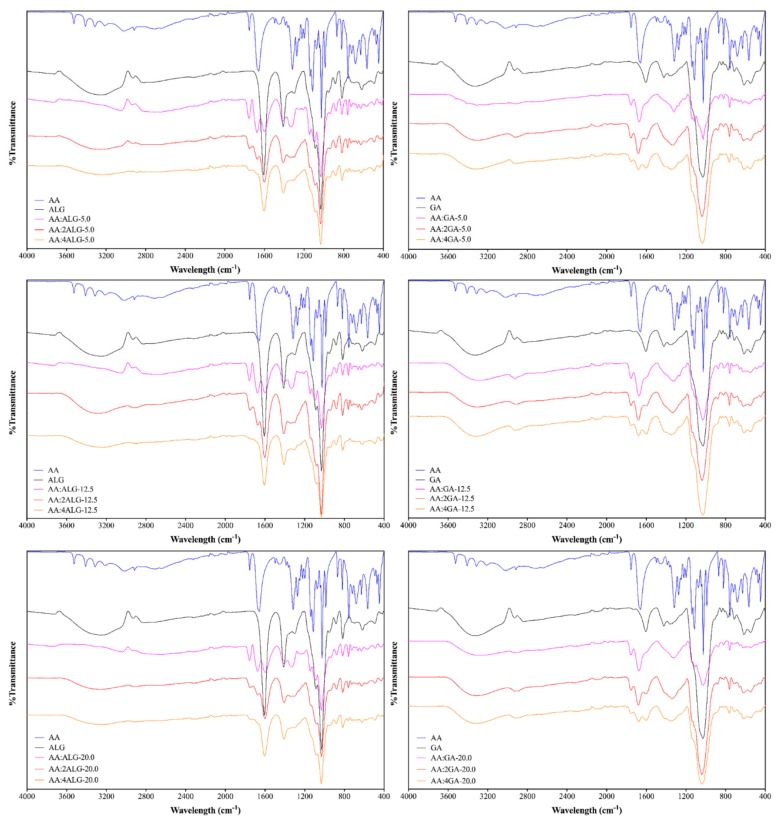
FTIR spectra of AA microparticles using ALG (left) and GA (right) as wall polymer.

The FTIR spectroscopy was employed to identify the different considered components and to evaluate the presence of the AA in the surface of the microcapsules. Figure 4 presents the spectra obtained for all AA:ALG and AA:GA-based systems compared with the control samples, while Table 4 gathers the major peaks and their band assignments between 400 at 4000 cm^−1^ for each component employed in the encapsulation process.

The pure AA spectra was assessed as reference, which revealed the characteristic absorption bands at 1658, 1750, and 3523 cm^−1^, corresponding to the presence of C=C, C=O, and O‒H stretching vibrations, respectively.

For the AA:ALG-based microparticles, the AA spectral peaks reduced their intensity as the polymer ratio increased, which suggested that AA was mainly covered by ALG. Indeed, in all the cases for the AA:4ALG composition, only the representative bands of the ALG polymer were perceived at 1030 cm^−1^ and 1606 cm^−1^ corresponding to the presence of C-O and C=O stretching vibrations.

However, for the AA:GA microparticles, the AA band corresponding to the C=O stretching vibrations remained in the spectra regardless the GA ratio employed. This observation may suggest that AA remains on the microparticles surface after the encapsulation process.

### 3.6. Thermo-Oxidative Stability

**Figure 5:**
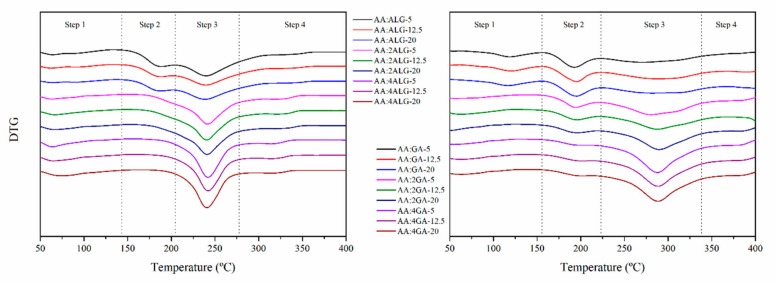
Derivative thermogravimetric curves of the AA:ALG (left) and AA:GA-based (right) microparticles.

The microparticles were characterised in terms of thermo-oxidative stability by means of thermogravimetric analysis (TGA) under an atmosphere of oxygen. For comparative purposes, the derivative thermo-gravimetric curves (DTG) were considered. The obtained DTG curves are shown in Figure 5, and the peak temperatures of the different stages along with the characteristic mass loss are gathered in Table 5 for the AA:ALG and AA:GA-based microparticles.

The thermo-oxidative decomposition of the pure components was initially assessed. The profile for the pure AA involved a three-stage process: (i) the main stage at 215 °C with a mass loss of 40%, followed by a stage (ii) at 306 °C and a 31% of mass loss and finally (iii) at 455 °C with a 29% of mass contribution. The pure ALG consisted in a three-stage process: (i) at 74 °C with a 15% of mass loss associated to the humidity release, (ii) at 245 °C with a mass contribution of 36%, and (iii) a stage at 625 °C with a 18% of mass loss correlated to the decomposition of the previously formed carbonaceous residue [51,52]. The pure GA revealed an analogous behaviour in accord with the thermogravimetric analysis described in Table 5; with (i) a bimodal humidity release stage with peaks at 72 and 103 °C and a mass loss of 9%, (ii) a main decomposition stage at 309 °C with 53% of mass loss and finally (iii) a decomposition at 513 °C with a mass loss of 27% [53,54].

For the microparticles, in general, a first decomposition stage was found from 50 to 150 °C due to the release of bound water molecules [51,55]. The mass-loss contribution increased from 6% to 10% as the ALG content increased in the AA:ALG-based microparticles and remained unaltered around 8% for the AA:GA samples. These results were according to those found in previous sections.

Considering the water release process, a four-stage decomposition behaviour was found for the AA:ALG composition, that turned into a three-stage comportment as the ALG content increased. For high ALG content, the decomposition processes associated to the AA progressively diminished and were overlapped by those of ALG. Moreover, the disappearance of the AA stage could be correlated to the protection against thermo-oxidative decomposition by means of encapsulation [56,57,58]. Moreover, the stage (iii) slightly moved towards higher temperatures and the stage (iv) towards lower temperatures, corroborating the ALG prevalence. For a given AA:ALG proportion, non-significant differences were perceived. Regarding the thermo-oxidative stability of the microparticles, they were stable up to 190 °C for the AA:ALG proportion and given the disappearance of the AA stage for the AA:2ALG and the AA:4ALG, the stability increased up to 240 °C.

For the AA:GA-based microparticles, a similar tendency was perceived. As the GA content increased, the contribution of the AA to the whole decomposition process significantly decreased and the mass loss percentage to the stage (iii) increased from 35% to 55% and moved towards higher temperatures. Moreover, considering the initial concentration of the AA, the calculated mass loss step during stage (ii) corroborated the pretended composition of the AA:GA-based microparticles. The increase in the total solid concentration prior to the spray-drying process slightly increased the stage (iii) temperature, but the global effect was not significant. From an overall perspective, although the thermo-decomposition process of the microparticles started at lower temperatures than the AA, they were stable up to 190 °C, which is above the traditional processing temperature for the pellet obtaining.

### 3.7. Thermal Properties

**Figure 6:**
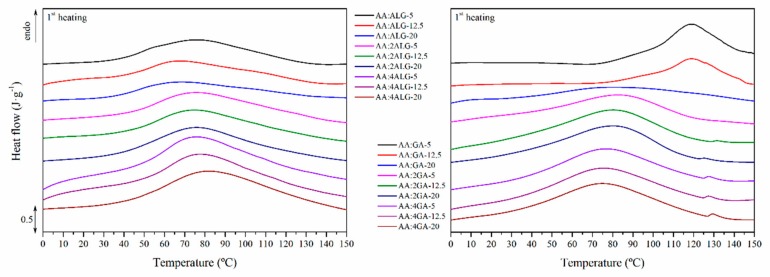
DSC first heating thermogram of the AA:ALG (left) and AA:GA-based (right) microparticles.

The thermal properties of the prepared microparticles were further evaluated by means of differential scanning calorimetry (DSC) to assess the thermal transitions in the range from 0 °C to 150 °C above the pellet processing temperature and below the beginning of the decomposition process. The obtained thermograms for the AA:ALG and AA:GA-based are plotted in Figure 6. The calculated temperatures and enthalpies are gathered in Table 6.

The pure AA revealed a flat thermogram in the analysed range. The pure ALG showed an endothermic transition during the first heating scan with a peak at 102 °C associated to a dehydration reaction [51,55]. Then, the glass transition was perceived around 110 °C [53,54]. The GA revealed a similar endothermic dehydration process in the first heating scan with a peak at 89 °C. Moreover, the glass transition around 90 °C was found in the subsequent cooling segment. The water release enthalpies were 233 and 167 J·g^−1^ for the ALG and GA, respectively, which may reflect the slight differences in the hydrophilicity of these materials.

In general, the water release process was perceived in all the analysed microparticles as a main and broad endothermic peak between 30 and 150 °C. On the one hand, the AA:ALG-based microparticles revealed a peak temperature for the water release between 67 and 82 °C. While the effect of the total solid content was irrelevant on this temperature, the increase in the ALG concentration slightly increased the peak temperature, given the stronger interactions within the water molecules. Moreover, the enthalpy associated to this transition increased as the ALG content did, which may suggest higher humidity retention, as found in the previous section. The glass transition was unperceivable on the AA:ALG-based microparticles.

On the other hand, the encapsulated AA:GA-based compositions revealed a water release temperature around 80 °C. The higher obtained values around 120 °C for the AA:GA-5 and AA:GA-12.5 may be due to its heterogeneous morphology. As described before, these compositions and total solid concentrations do not allow for the microparticles obtaining. The water release enthalpy also remained constant around 160 J·g^−1^ in all the cases, given the similar moisture content reported before. Regarding the glass transition, it was slightly perceived in the second heating scan at 80 °C, which was overlapped by the water release process.

## 4. Conclusions

Microparticles containing AA with a mean diameter around 6.0 μm and 9.1 μm and an average product yield of 57% and 74% were obtained using ALG and GA, respectively. For both biopolymers evaluated, AA encapsulation efficiency above 90% was obtained. The AA microparticles exhibited a spherical geometry and a regular shape without fissures. Nevertheless, a high solid dispersed concentration is necessary to obtain a spherical geometry when GA is employed.

The encapsulation of AA with sodium alginate and gum Arabic using the spray-drying technique potential wall avoids thermo-oxidative degradation. The ALG and GA biopolymers maintain AA stable up to 188 °C, which is higher than the traditional processing temperature used for the fish feed industry (100 to 120 °C). For high ALG content, the AA thermogravimetric decomposition stage disappearance, presumably by the protection of wall polymer as a result of the microcapsule formation.

## Figures and Tables

**Table 1:** Taguchi L_9_ orthogonal array for L-ascorbic acid encapsulation formulation.

AA:Polymer Ratio	Total Dispersed Solids (g·L^−1^)	AA:ALG Label	AA:GA Label
1:1	5.0	AA:ALG-5	AA:GA-5
1:1	12.5	AA:ALG-12.5	AA:GA-12.5
1:1	20.0	AA:ALG-20	AA:GA-20
1:2	5.0	AA:2ALG-5	AA:2GA-5
1:2	12.5	AA:2ALG-12.5	AA:2GA-12.5
1:2	20.0	AA:2ALG-20	AA:2GA-20
1:4	5.0	AA:4ALG-5	AA:4GA-5
1:4	12.5	AA:4ALG-12.5	AA:4GA-12.5
1:4	20.0	AA:4ALG-20	AA:4GA-20

**Table 2:** AA encapsulation efficiency (%), mean particle size (µm) encapsulation yield (%) and moisture (%) for AA:ALG and AA:GA-based microparticles.

		Encapsulation Efficiency (%)	Mean Particle Size (µm)	Encapsulation Yield (%)	Moisture (%)
AA:ALG-based	AA:ALG-5	97.9 ± 1.9	5.1 ± 0.4	75.7 ± 0.8	2.5 ± 0.1
	AA:ALG-12.5	98.9 ± 1.1	10.9 ± 1.2	63.1 ± 0.2	2.1 ± 0.3
	AA:ALG-20	98.0 ± 1.6	11.0 ± 2.4	42.0 ± 1.1	2.6 ± 1.0
	AA:2ALG-5	90.5 ± 2.8	8.2 ± 1.2	70.8 ± 0.7	3.9 ± 2.3
	AA:2ALG-12.5	93.9 ± 4.4	7.8 ± 1.0	62.1 ± 0.5	4.3 ± 2.5
	AA:2ALG-20	93.0 ± 2.4	7.8 ± 0.4	35.2 ± 6.7	4.0 ± 2.6
	AA:4ALG-5	92.2 ± 2.8	9.7 ± 0.7	67.1 ± 3.0	4.1 ± 1.4
	AA:4ALG-12.5	96.3 ± 2.3	7.6 ± 0.2	63.6 ± 0.4	5.5 ± 3.0
	AA:4ALG-20	92.6 ± 1.8	14.1 ± 1.4	37.8 ± 1.4	4.3 ± 2.2
AA:GA-based	AA:GA-5	95.0 ± 4.4	9.5 ± 2.3	61.1 ± 6.7	2.4 ± 1.7
	AA:GA-12.5	98.3 ± 1.2	7.8 ± 0.6	68.5 ± 1.8	1.4 ± 0.5
	AA:GA-20	82.6 ± 4.3	9.7 ± 0.5	67.8 ± 4.4	1.6 ± 0.8
	AA:2GA-5	96.8 ± 3.4	2.9 ± 0.2	79.3 ± 1.1	5.0 ± 2.0
	AA:2GA-12.5	98.4 ± 0.4	4.0 ± 0.1	83.2 ± 1.0	4.4 ± 2.8
	AA:2GA-20	89.6 ± 5.6	5.3 ± 0.4	75.6 ± 3.3	4.5 ± 2.8
	AA:4GA-5	91.6 ± 0.4	3.1 ± 0.5	78.4 ± 1.0	5.4 ± 2.7
	AA:4GA-12.5	96.0 ± 3.0	5.3 ± 0.0	77.8 ± 4.0	5.4 ± 2.6
	AA:4GA-20	93.8 ± 2.8	6.3 ± 0.9	77.0 ± 0.9	4.8 ± 2.7

**Table 3:** Regression model predicted coefficients and ANOVA analysis for the AA encapsulation employing ALG and GA as wall polymers. The acronyms C1 and C2 correspond to coefficients of responses for AA:polymer ratio and total solid dispersed respectively.

		Predicted Factors by Taguchi						ANOVA	
		Constant	C1	C2	C1C1	C2C2	R^2^	Ratio	SD
**AA:ALG**	Encapsulation efficiency	94.02	−2.26	0.54	3.50	−2.35	0.67	54.74	12.73
	Mean particle size	7.57	0.72	1.66	1.79	0.53	0.44	15.75	28.34
	Encapsulation yield	61.45	−2.04	−16.43	2.20	−8.17	0.96	1.88	95.05
**AA:GA**	Encapsulation efficiency	98.91	0.91	−2.88	−2.04	−5.98	0.53	5.26	47.94
	Mean particle size	3.80	−2.06	0.98	2.88	0.41	0.86	74.98	10.80
	Encapsulation yield	77.74	7.71	−0.89	−6.74	−1.15	0.79	77.86	1.29

**Table 4:** Positions of major IR stretching bands and their respective assignments for AA, ALG and GA.

Characteristic Group	Wavelength (cm^−1^)		
	AA	ALG	GA
C—O stretch	1025	1030	1029
C—O—C stretch	1113	1338	–
C=C stretch	1658	–	–
C=O stretch	1750	1606	1604
C—H stretch	3350	2932	2910
O—H stretch	3523	3260	3333
C(=O)—O stretch	–	1411	1422
(1,4), (1,6) linkage of galactose and mannose	–	–	614

**Table 5:** Peak temperatures and mass loss during the thermogravimetric analyses for the AA:ALG and AA:GA-based microparticles.

		Stage (i) (H_2_O)		Stage (ii) (AA)		Stage (iii) (ALG + AA) (GA + AA)		Stage (iv) (Char)	
		T_1_	Step_1_	T_2_	Step_2_	T_3_	Step_3_	T_4_	Step_4_
		(°C)	(%)	(°C)	(%)	(°C)	(%)	(°C)	(%)
**AA:ALG-based**	AA:ALG-5	64.20	7.45	188.90	20.86	239.95	38.98	335.88	5.78
	AA:ALG-12.5	62.30	5.38	188.00	13.33	239.50	34.22	337.51	5.61
	AA:ALG-20	63.74	5.28	187.85	12.62	238.82	30.48	336.70	5.33
	AA:2ALG-5	63.07	6.38	–	–	241.54	38.10	318.36	5.52
	AA:2ALG-12.5	66.58	7.71	–	–	240.41	38.17	320.34	5.68
	AA:2ALG-20	66.04	8.25	–	–	240.96	41.86	318.04	6.85
	AA:4ALG-5	64.10	8.95	–	–	241.81	41.60	313.29	5.21
	AA:4ALG-12.5	65.04	10.22	–	–	242.09	40.60	313.75	5.57
	AA:4ALG-20	74.12	10.97	–	–	240.65	41.73	313.94	5.21
AA:GA-based	AA:GA-5	*55.5*	8.43	192.59	20.44	269.89	34.39	377.88	12.04
	AA:GA-12.5	*51.97*	8.65	195.35	18.83	288.86	38.48	388.11	12.02
	AA:GA-20	*49.43*	8.37	195.27	18.96	283.09	39.19	400.53	14.02
	AA:2GA-5	56.96	7.38	194.54	16.83	280.77	46.35	378.49	9.26
	AA:2GA-12.5	63.17	7.08	195.78	15.56	287.69	43.19	404.78	15.34
	AA:2GA-20	52.35	7.32	196.17	14.55	289.56	48.38	380.36	11.08
	AA:4GA-5	54.28	7.19	202.84	11.96	287.71	55.13	377.86	7.16
	AA:4GA-12.5	62.82	8.20	202.92	13.48	288.41	54.59	379.11	9.46
	AA:4GA-20	64.19	7.75	202.44	12.75	296.93	55.06	377.27	7.79

**Table 6:** Peak temperatures and enthalpies of the first heating thermogram for the AA:ALG and AA:GA-based microparticles.

AA:ALG Label	T (°C)	∆H (J·g^−1^)	AA:GA Label	T (°C)	∆H (J·g^−1^)
AA:ALG-5	76.04	101.74	AA:GA-5	118.49	117.71
AA:ALG-12.5	67.59	97.10	AA:GA-12.5	119.27	121.63
AA:ALG-20	68.81	91.85	AA:GA-20	81.88	151.69
AA:2ALG-5	76.44	140.08	AA:2GA-5	82.31	158.95
AA:2ALG-12.5	75.77	143.02	AA:2GA-12.5	80.13	161.66
AA:2ALG-20	75.98	145.68	AA:2GA-20	80.46	160.57
AA:4ALG-5	76.13	172.91	AA:4GA-5	79.30	156.14
AA:4ALG-12.5	77.56	159.65	AA:4GA-12.5	79.32	151.23
AA:4ALG-20	82.33	197.84	AA:4GA-20	78.67	154.86

## Supplementary Materials

The following are available online at https://www.mdpi.com/1420-3049/24/16/2872/s1, Table S1: ANOVA analysis for the response variables: Encapsulation efficiency(%), mean particle size (µm) and encapsulation yield (%) involved in the AA encapsulation using Sodium Alginate and Gum Arabic as wall polymers, Table S2: Water activity for the AA microparticles obtained using ALG and GA as wall polymers.
