# Supplementary material for: Spray-Drying Performance and Thermal Stability of L-ascorbic Acid Microencapsulated with Sodium Alginate and Gum Arabic
Source: Molecules. 2019 Aug 7;24(16):2872. doi: 10.3390/molecules24162872 (PMC6721127; doi:10.3390/molecules24162872)
Supplement: Supplementary file 1 [file molecules-24-02872-s001.pdf]

Supplementary Materials

Spray-Drying Performance and Thermal Stability of L-Ascorbic Acid Microencapsulated with Sodium Alginate and Gum Arabic

Pabla A. Barra [1], Katherine Márquez [1], Oscar Gil-Castell [2,3], Javiera Mujica [4], Amparo Ribes-Greus [3] and Mirko Faccini [1,5,*]

[1] R&D Department, Leitat Chile, Calle Román Díaz 532, Providencia, Santiago 7500724, Chile
[2] Instituto de Tecnología de Materiales (ITM), Universitat Politècnica de València (UPV), Camino de Vera s/n, 46022 Valencia, Spain
[3] Departament d'Enginyeria Química, Escola Tècnica Superior d'Enginyeria, Universitat de València, Av. de la Universitat, s/n, 46100 Burjassot, Spain
[4] Centro de Excelencia en Nanotecnología (CEN) Chile, Calle Román Diaz 532, Providencia, Santiago 7500724, Chile
[5] Materials Chemistry Division, Leitat Technological Center, C/Pallars 179–185, 08005 Barcelona, Spain
* Correspondence: mfaccini@leitat.org

Table 1. ANOVA analysis for the response variables: Encapsulation efficiency (%), mean particle size (µm) and encapsulation yield (%) involved in the AA encapsulation using Alginic acid and arabic gum as wall polymers.

	Factor	DOF (f)	Sum of squares	F-ratio (F)	p-Value
AA:ALG-based	**i) Encapsulation Efficiency (%)**				
	AA:ALG ratio	2	127.46	10.94	0.002
	Dispersed solids (g/L)	2	29.63	2.54	0.117
	Error	13	75.72		
	Total	17	232.81		
	**ii) Mean particle size (µm)**				
	AA:ALG ratio	2	19.1	1.83	0.199
	Dispersed solids (g/L)	2	34.3	3.29	0.070
	Error	13	67.71		
	Total	17	121.11		
	**iii) Encapsulation Yield (%)**				
	AA:ALG Ratio	2	69.6	3.99	0.044
	Dispersed solids (g/L)	2	3508.6	201.52	1.64E-10
	Error	13	113.17		
	Total	17	3691.37		
AA:GA-based	**i) Encapsulation Efficiency (%)**				
	AA:GA ratio	2	30.77	0.73	0.500
	Dispersed solids (g/L)	2	280.32	6.56	0.010
	Error	13	273.69		
	Total	17	584.78		
	**ii) Mean particle size (µm)**				
	AA:GA ratio	2	84.14	34.27	6.55E-06
	Dispersed solids (g/L)	2	12.12	4.94	0.025
	Error	13	15.96		
	Total	17	112.22		

**iii) Encapsulation Yield (%)**				
AA:GA ratio	2	894.8	24.28	4.10E-05
Dispersed solids (g/L)	2	14.9	0.40	0.68
Error	13	239.55		
Total	17	1149.22		

Table 2. Water Activity for the AA microparticles obtained using ALG and GA as wall polymers.

	**AA:ALG**	**Water Activity (a_w)**	**T (°C)**
**AA:ALG-based**	AA:ALG-5	0.27 ± 0.01	24.8
	AA:ALG-12.5	0.38 ± 0.06	25.0
	AA:ALG-20	0.34 ± 0.01	24.9
	AA:2ALG-5	0.35 ± 0.01	24.9
	AA:2ALG-12.5	0.35 ± 0.02	24.8
	AA:2ALG-20	0.36 ± 0.02	24.8
	AA:4ALG-5	0.35 ± 0.01	25.1
	AA:4ALG-12.5	0.32 ± 0.01	25.1
	AA:4ALG-20	0.37 ± 0.01	25.7
	AA:ALG-5	0.30 ± 0.01	25.9
**AA:GA-based**	AA:GA-5	0.39 ± 0.03	25.0
	AA:GA-12.5	0.26 ± 0.01	24.9
	AA:GA-20	0.23 ± 0.02	24.6
	AA:2GA-5	0.23 ± 0.01	24.6
	AA:2GA-12.5	0.32 ± 0.01	24.9
	AA:2GA-20	0.20 ± 0.01	24.6
	AA:4GA-5	0.21 ± 0.02	24.7
	AA:4GA-12.5	0.23 ± 0.01	24.9
	AA:4GA-20	0.23 ± 0.01	24.9
